# Brace‐related Stress and Quality of Life Parameters between Chêneau and Boston Braces: A Cross‐sectional Comparative Study on Adolescent Idiopathic Scoliosis in Saudi Arabia

**DOI:** 10.1111/os.14121

**Published:** 2024-06-10

**Authors:** Abdulmonem M. Alsiddiky, Khalid S. Alharbi, Omar A. Ababtain, Abdullah F. Alnuwaybit, Mazin A. Zamzami, Ahmad A. Basalah, Wesam H. Al‐Sabban

**Affiliations:** ^1^ Department of Orthopedic Surgery, College of Medicine, Research Chair of Spinal Deformities King Saud University Riyadh Saudi Arabia; ^2^ College of Medicine King Saud University Riyadh Saudi Arabia; ^3^ Biochemistry Department, Faculty of Science, Centre of Artificial Intelligence for Precision Medicines King Abdulaziz University Jeddah Saudi Arabia; ^4^ Mechanical Engineering Department, College of Engineering and Islamic Architecture Umm Al Qura University Makkah Saudi Arabia; ^5^ Department of Information Systems, College of Computer and Information Systems Umm Al‐Qura University Makkah Saudi Arabia

**Keywords:** Adolescent, Braces, Deformity, Quality of life, Satisfaction, Scoliosis

## Abstract

**Objective:**

Adolescent idiopathic scoliosis (AIS) is the most prevalent spinal deformity affecting healthy children. Although AIS typically lacks symptomatic manifestations, its resultant deformities can affect patients' quality of life (QoL). Evaluating QoL and stress levels is crucial in determining the optimal brace type for AIS patients; however, research comparing the effectiveness of different brace types in this regard is lacking. Therefore, this study aimed to evaluate the impact of Boston versus Chêneau braces on QoL and stress levels in AIS patients.

**Methods:**

This cross‐sectional study was conducted at a medical institution in Riyadh, Saudi Arabia, involving 52 eligible patients selected through stratified random sampling based on type of brace as the main stratum. The inclusion criteria were idiopathic scoliosis, age ≥ 10 years, bracing for at least 3 months, and no history of cancer. QoL was evaluated according to the revised Scoliosis Research Society 22‐item questionnaire (SRS‐22r) and stress levels according to the eight‐item Bad Sobernheim stress questionnaire (BSSQ‐Brace). Independent‐sample *t*‐tests were used to compare brace‐related QoL and stress level according to participants' sex and brace type.

**Results:**

Overall, 32 participants were treated with Boston braces (seven men and 25 women), with a median (IQR) age of 11.00 years (10.00–13.00), and 20 participants were treated with Chêneau braces (three men, 17 women), with a median (IQR) age of 12.50 years (10.00–14.25). The total SRS‐22 score was not significantly different between the brace groups (*p* = 0.158). However, patients in the Boston brace group reported significantly higher satisfaction levels (median = 4.00, IQR = 3.50–4.50) than did those in the Chêneau brace group (median = 3.25, IQR = 2.38–4.13, *p* = 0.013, moderate effect size = 0.345, 95% CI = 0.060 to 0.590). Furthermore, the BSSQ‐brace total score was significantly higher in the Boston brace group (median = 9.00, IQR = 8.00–12.00) than in the Chêneau brace group (median = 7.50, IQR = 4.75–10.00, *p* = 0.007, moderate effect size = 0.376, 95% CI = 0.130 to 0.590), indicating higher stress levels in the Chêneau brace group.

**Conclusion:**

The QoL in AIS patients undergoing brace treatment was comparable across groups. Nonetheless, patients who used Chêneau braces experienced higher stress levels and lower treatment satisfaction rates than did those who used Boston braces. These findings can inform clinical decisions regarding prescription of bracing types and highlight the need for further in‐depth research.

## Graphical abstract

This study reveals that gender‐based disparities have no significant impact on stress and quality of life in adolescent idiopathic scoliosis (AIS) patients undergoing brace treatment. Notably, it underscores that Chêneau brace users tend to report lower satisfaction and heightened stress levels, offering valuable insights for spinal surgeons and advancing research in this field.

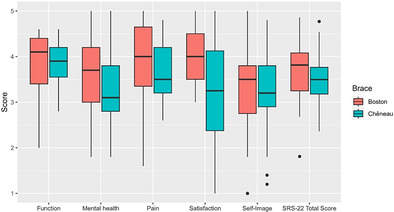

## Introduction

Adolescent idiopathic scoliosis (AIS) is the most prevalent spinal deformity in healthy youngsters, affecting approximately 0.5%–5.2% of children aged 10–16 years globally.^1^ In Saudi Arabia, the national school‐based screening program, aimed at screening and monitoring the health of school‐aged children, has estimated the prevalence of scoliosis at 0.48%.^2^ Although many forms of scoliosis exist, most of the scoliosis cases (84%–89%) are reported in previously healthy teenagers during the adolescent growth spurt.^3^ Despite often being asymptomatic and non‐life‐threatening, the resultant deformities of AIS can significantly affect patients' quality of life (QoL),^4^ eliciting concerns about physical appearance, pain, and functional limitations.^1^

The current management approach for AIS encompasses both surgical and conservative treatment. Conservative treatments include orthotic spinal bracing, exercise, and clinical monitoring.^5^ In particular, bracing emerges as the most common conservative treatment for AIS patients, aimed at limiting further spinal curve progression.^1^ Bracing is the process of applying external supports to the trunk to achieve the greatest possible correction of abnormal spinal curve.^6^ Treatment usually begins when the curve is progressing or when the Cobb angle exceeds a threshold of 30°.^7^

The gold standard is bracing for 18–20 h or more per day using a rigid thoracolumbosacral orthosis (TLSO).^8^ There are two primary categories: passive braces, which aim to hold the spine in a straighter position, and active braces, which apply forces to the body to promote realignment of the spine.^9^ Types of scoliosis braces include the Milwaukee brace, one of the first full‐torso braces that extends from the neck to the pelvis; the Boston brace, a lower‐profile TLSO that fits under clothing; and the Chêneau brace, a custom‐made TLSO that encourages guided growth through asymmetric pressure.^10^ Among the various bracing options, the Boston brace is one of the most popular braces for scoliosis patients.^11^ Featuring an open back design, it utilizes small pads to apply pressure to the spine, thereby limiting scoliosis progression.^12^ In contrast, a Chêneau‐style brace incorporates three‐dimensional biomechanical forces, specifying where to construct pads and where to install expansion rooms designed to oppose spinal torsion and correct scoliosis.^12^

While orthotic treatment has been shown to be effective in stopping curve progression in moderate AIS cases (Cobb angle: 20°–40°), its effectiveness depends on various variables, such as orthotic design and patient compliance.^13^ Previous studies have shown that use of a brace in patients with AIS negatively affects their QoL and has psychological effects, including negative thoughts, stress, anxiety, emotional instability, and disturbed self‐image or self‐esteem.^5^, ^7^, ^14^, ^15^ Moreover, poor compliance is associated with low QoL, leading to treatment failure and patient dissatisfaction.^16^

Despite these findings, direct comparisons of QoL among different brace types are scarce. Given that duration of brace wear is a valuable predictor of treatment outcomes, factors associated with deterioration of QoL should be addressed, to increase brace treatment tolerance and comfort in patients with AIS, particularly in the context of Saudi Arabia.^17^ This study addresses this gap by investigating the QoL differences between patients using the Boston brace, and the Chêneau brace.^10^ This will help for better clinical decision‐making and enhancing treatment adherence for such patients in Saudi Arabia.

## Methods

### Study Design

A survey instrument was meticulously developed to collect feedback from patients with AIS who have undergone brace treatment. The survey collected demographic information (such as age and sex), initial Cobb angle measurement at treatment initiation, and treatment trajectory. Furthermore, the survey assessed patient satisfaction with their current brace, level of physical activity, and psychological stress associated with brace use. A cross‐sectional sampling method was employed, encompassing users of Boston and Chêneau braces. In our survey, we evaluated QoL using the revised Scoliosis Research Society 22‐item questionnaire (SRS‐22r) and assessed stress levels using the eight‐item Bad Sobernheim stress questionnaire (BSSQ‐Brace). A total of 60 patients were invited to complete the survey, of which 52 responses were received, with a response rate of 87%.

### Study Setting

The study was conducted in Riyadh, the capital of Saudi Arabia, at a single medical institution between February 2023 and June 2023, with 52 patients of varying educational, financial, and cultural backgrounds, contributing to diversity of the sample. The study participants were male and female patients with AIS who underwent brace treatment with either the Boston or Chêneau models. Recruited patients were contacted using their details obtained from the orthopedic clinics that they regularly attended and followed up with. Subsequently, each patient was individually contacted by a verified healthcare provider and asked to participate in the survey, thereby ensuring a personalized and tailored approach to data collection. Participants were selected based on the inclusion criteria recommended by the Scoliosis Research Society (SRS) and the International Society on Scoliosis Orthopedic and Rehabilitation Treatment (SOSORT).^18^, ^19^ Multiple criteria were applied for participant inclusion in the study including an age of 10 years or older when brace is prescribed, primary curve angles 25°–40°, no prior treatment, ongoing treatment for at least 3 months, and, if female, either pre‐menarche or less than 1 year post menarche. Patients with non‐idiopathic scoliosis were excluded from the study. Furthermore, wearing braces were checked during the follow‐up visits, patients who reported wearing the brace for less than 23 hours per day were also excluded. A thorough review of each patient's medical records was conducted to minimize recall bias and verify their eligibility for the study. A comprehensive assessment of their suitability for participation in the study was undertaken.

### Sampling Technique and Data Collection

Stratified random sampling was performed to compare stress levels and QoL parameters between the two groups, with type of brace used as the main stratum. To evaluate the effect of treatment duration on stress levels and QoL in our study sample, patients were divided into the following four groups: patients who had worn a brace for <6 months (n = 15), those who had worn a brace <12 months (n = 9), those who had worn a brace <24 months (n = 14), and those who had worn a brace >24 months (n = 14). All braces were made for each patient by a qualified orthotist, and the patients were asked to wear their braces for 23 hours/day. All patients completed the Arabic version of the revised SRS‐22r and the eight‐item BSSQ‐Brace.

The SRS‐22r is a disease‐specific questionnaire designed to assess patient‐reported outcomes. It consists of five different sections that assess: (i) function/activity; (ii) pain; (iii) self‐image/appearance; (iv) mental health; and (v) treatment satisfaction. Each section comprises five items, other than the “satisfaction with the treatment” section, which had only two items. Responses were recorded on a five‐point Likert scale, ranging from 1 (worst) to 5 (best), with scores for each section ranging from 5 to 25 (other than the “satisfaction with the treatment” section, for which the score ranged from 2 to 10). The total score range was 22–110 points, with higher scores indicated a better QoL. Participants with AIS were required to answer all questions on the SRS‐22r questionnaire.^20^

While it is recognized that the SRS‐22r is traditionally used in the context of surgical interventions, its applicability has been substantiated in conservative treatment settings as well. The SRS‐22r offers a comprehensive assessment of health‐related QoL regardless of treatment modality.^21^ Studies have corroborated the sensitivity of the SRS‐22r in detecting changes in QoL among patients treated with braces, thereby validating its use outside of surgical contexts.^22^, ^23^ Furthermore, the questionnaire is backed by extensive validation processes across different patient groups, ensuring its reliability and construct validity for assessing quality of life in scoliosis treatment.^24^ The utilization of the SRS‐22r in our study was thus a strategic decision to enrich the data on the impact of bracing on adolescents and to facilitate the enhancement of clinical decision‐making and patient care.

Brace‐related stress was evaluated using the brace version of the BSSQ. The brace version focuses on the brace‐related mood, social interactions, and subsequent stress levels. It consists of eight items scored on a four‐point Likert scale, ranging from 0 (indicating the highest level of stress) to 3 (indicating the lowest level of stress). The total score on the questionnaire ranges from 0 to 24 and can be classified into three levels based on the stress level: a score of 0–8 indicating a high stress level, 9–16 indicating moderate stress level, and 17–24 indicating low stress level.^15^

All participants in the two groups were required to answer both the BSSQ‐Brace and SRS‐22r questionnaires. The analysis focused exclusively on complete questionnaires, thereby excluding incomplete questionnaires or missing data from consideration. To ensure validity and reliability of the study findings, several strategies were implemented to address potential biases. To mitigate the influence of recall bias, a comprehensive and meticulous review of electronic medical records was conducted for each patient. This approach aimed to gather accurate and detailed information, minimizing the reliance on participants' memory recall. Additionally, to tackle the potential influence of response bias, a thorough examination of participant responses was conducted. This involved systematic scrutiny of data to identify any discernible patterns or indications of careless completion, ensuring reliability and credibility of the collected responses, and leading to more robust research outcomes. For confounding variables, a rigorous selection procedure was implemented. Specifically, patients enrolled in the study were carefully chosen to have no previous or concurrent medical or surgical history, other than AIS. Furthermore, utmost attention was given to achieving comparability between the groups in terms of scoliosis severity and compliance. This was done to minimize the potential impact of confounding factors and bolster the internal validity of the research findings.

### Ethical Considerations

This study involving human participants was conducted in accordance with the Declaration of Helsinki and approved by our Institutional and/or National Research Committee (IRB Project No. E‐23‐7548). The participants and their legal guardians were given a clear explanation of the study's purpose and informed that their participation was voluntary, with the right to decline participation. Informed consent for study participation was obtained from all the participants and their legal guardians. To ensure anonymity, each participant was assigned a code number for the analysis.

### Statistical Analysis

**FIGURE 1:**
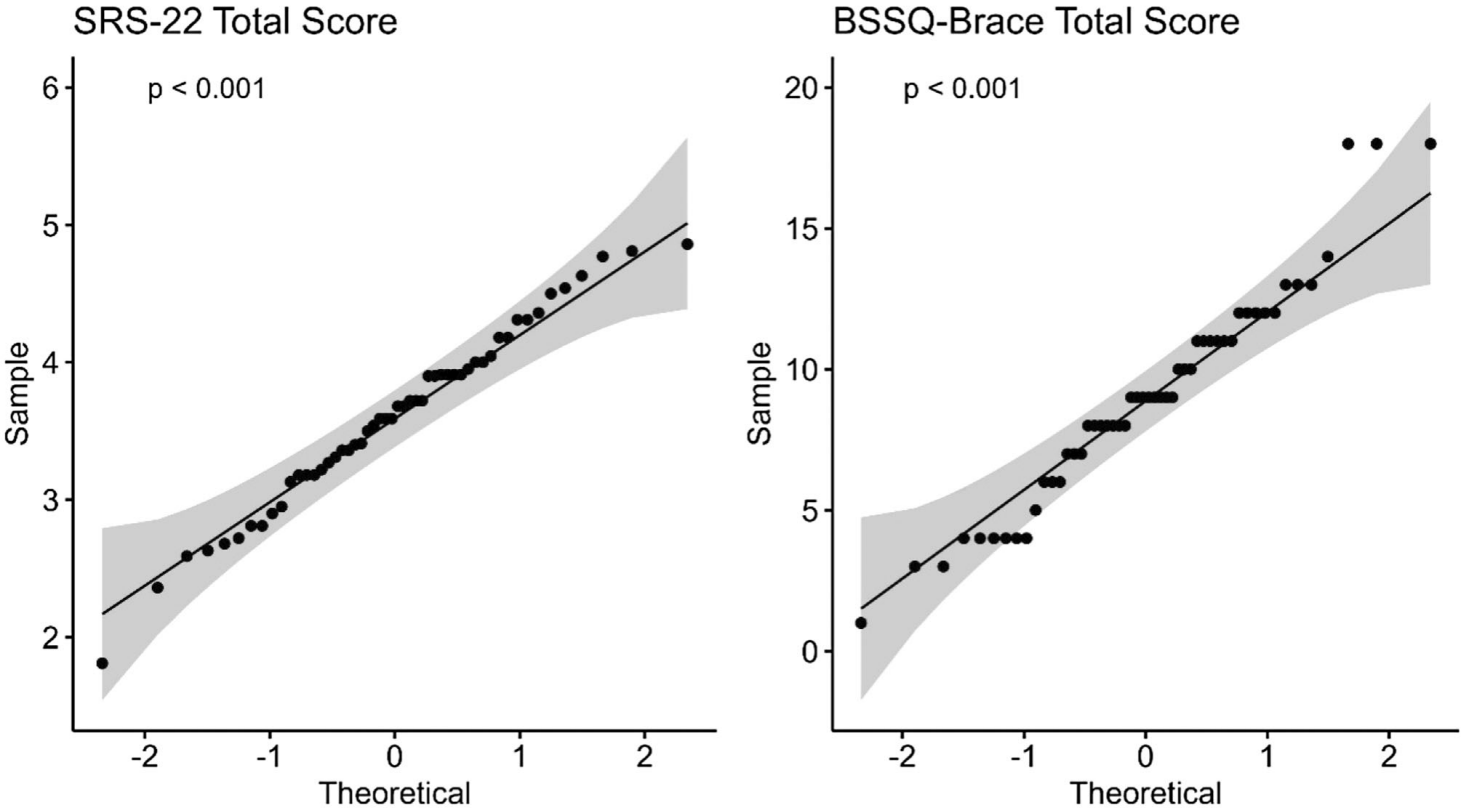
Results of normality testing for numerical variables under investigation. *P* values are those resulting from the Shapiro–Wilk test.

Data analysis was performed using RStudio (R version 4.3.1.). Normality testing was performed for continuous variables, including the SRS‐22r and BSSQ scores. This included the Shapiro–Wilk test and visualization of the QQ plots. Results revealed that both the SRS‐22r and BSSQ scores were non‐normally‐distributed (*p* < 0.001 for both), which was corroborated by the abnormal distribution illustrated in Figure 1. Therefore, we present numerical variables as medians and interquartile ranges (IQRs) and categorical variables as frequencies and percentages. Non‐parametric tests were applied in the analysis. Differences in numerical variables were assessed using a Wilcoxon rank sum test for variables with two categories or a Kruskal–Wallis rank sum test for variables with more than two categories. Effect sizes were created based on an independent two‐sample test (Wilcoxon rank sum test), and the values were interpreted as follows: a small effect at 0.10 to <0.30, a moderate effect at 0.30 to 0.50, and a large effect at 0.50 or more. Effect sizes are demonstrated with their respective 95% confidence interval (CI). Fisher's exact test was used to explore differences in proportions between categorical variables.

## Results

### Characteristics of the Study Population

Among the 52 included patients, 32 were treated with the Boston brace (seven men, 25 women), with a median (IQR) age of 11.00 years (10.00–13.00), and 20 were treated with the Chêneau brace (three men, 17 women), with a median (IQR) age of 12.50 years (10.00–14.25). The median (IQR) Cobb angle at treatment initiation was 29.65° (24.53°–34.05°) in the Boston brace group and 31.45° (27.50°–37.85°) in the Chêneau brace group. No significant differences were noted between the brace groups in terms of sex, age, and Cobb angle (Table 1).

**TABLE 1:** Characteristics of the study population.

Characteristic	Boston N = 32	Chêneau N = 20	*p*‐value
Sex			0.722
Male	7 (21.9%)	3 (15.0%)	
Female	25 (78.1%)	17 (85.0%)	
Age (years)	11.00 (10.00–13.00)	12.50 (10.00–14.25)	0.178
Cobb angle (°)	29.65 (24.53–34.05)	31.45 (27.50–37.85)	0.314
Brace wearing time (months)			0.226
<6	12 (37.5%)	3 (15.0%)	
<12	6 (18.8%)	3 (15.0%)	
<24	8 (25.0%)	6 (30.0%)	
>24	6 (18.8%)	8 (40.0%)	

*Note*: Data are presented as n (%) or median (IQR).

### QoL and Stress Levels in the Boston and Chêneau Brace Groups

**FIGURE 2:**
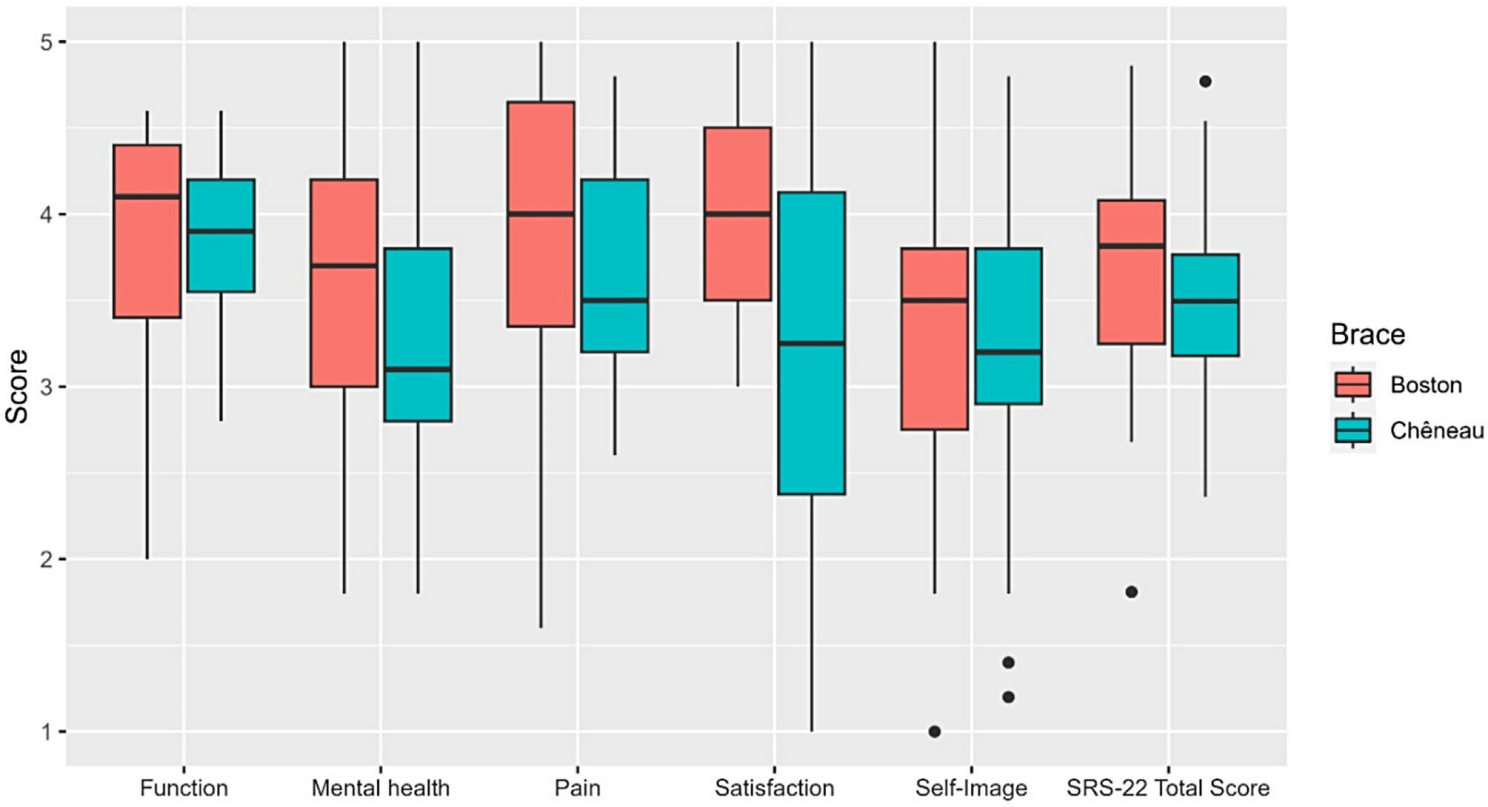
Boxplots showing scores of each domain of the SRS‐22r questionnaire in patients treated with the Boston and Chêneau braces. SRS‐22r, Scoliosis Research Society 22‐item questionnaire.

**FIGURE 3:**
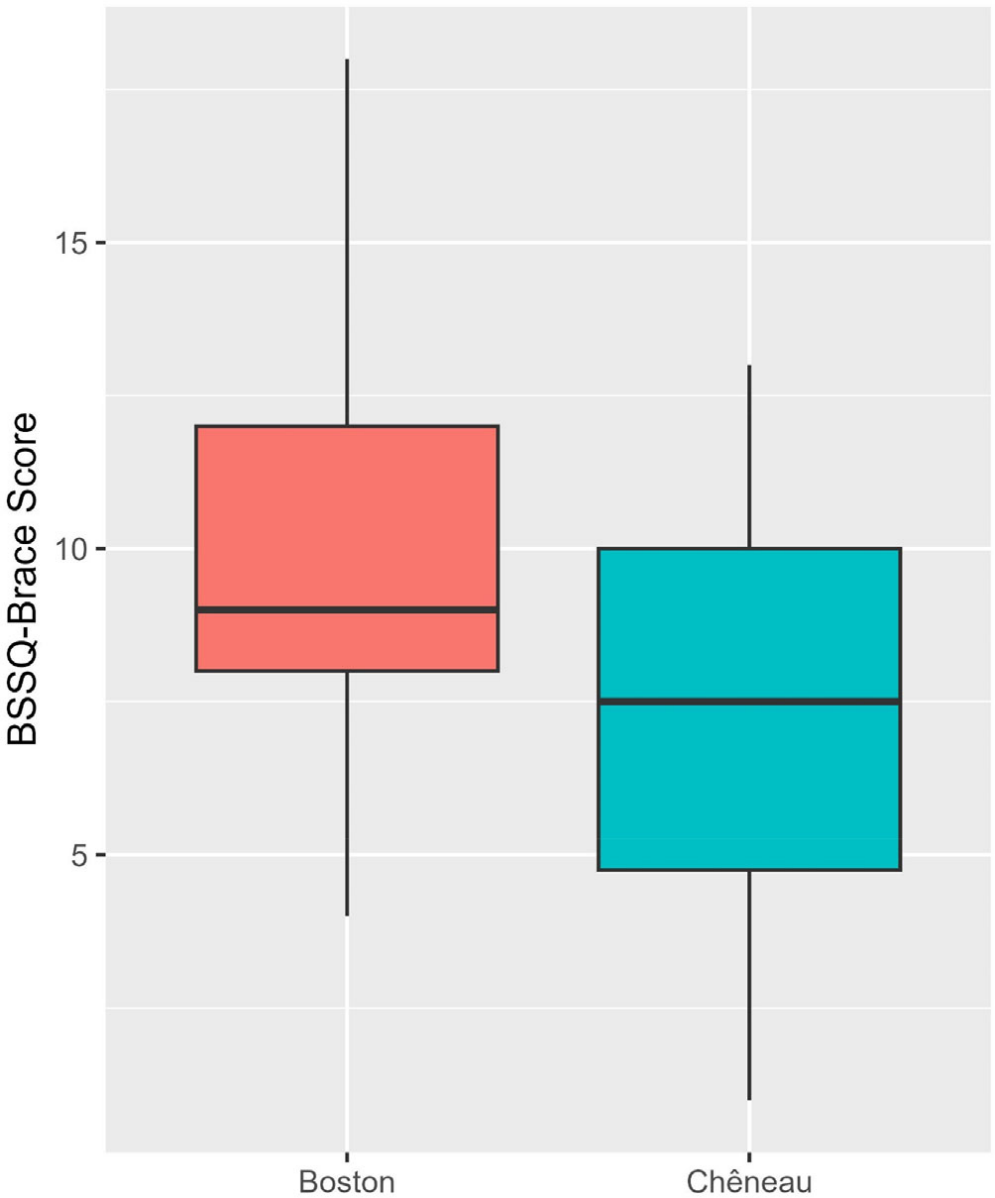
Boxplots showing the BSSQ scores in patients treated with the Boston and Chêneau braces.

Comparison between the Boston and Chêneau brace groups revealed significant differences in several variables (Table 2 and Figure 2). The total SRS‐22 score was not significantly different between the groups (*p* = 0.158). However, in terms of subdomains, patients in the Boston brace group reported significantly higher treatment satisfaction level (median = 4.00, IQR = 3.50–4.50) than those in the Chêneau brace group (median = 3.25, IQR = 2.38–4.13, *p* = 0.013, moderate effect size = 0.345, 95% CI = 0.060 to 0.590). Other subdomains, including function (*P* = 0.872), pain (*p* = 0.253), self‐image (*p* = 0.651), and mental health (*p* = 0.203) did not exhibit significant differences between the two groups. The BSSQ‐Brace total score was significantly higher in the Boston brace group (median = 9.00, IQR = 8.00–12.00) than in the Chêneau brace group (median = 7.50, IQR = 4.75–10.00, *p* = 0.007, moderate effect size = 0.376, 95% CI = 0.130 to 0.590, Table 2 and Figure 3).

**TABLE 2:** Comparison of QoL and stress levels between the Boston and Chêneau brace groups

Characteristic	Boston N = 32	Chêneau N = 20	*p*‐value	Effect size	Magnitude
Function	4.10 (3.40–4.40)	3.90 (3.55–4.20)	0.872	0.024 (0.004 to 0.300)	Small
Pain	4.00 (3.35–4.65)	3.50 (3.20–4.20)	0.253	0.160 (0.008 to 0.410)	Small
Self‐image	3.50 (2.75–3.80)	3.20 (2.90–3.80)	0.651	0.064 (0.005 to 0.340)	Small
Mental health	3.70 (3.00–4.20)	3.10 (2.80–3.80)	0.203	0.178 (0.010 to 0.450)	Small
**Satisfaction**	**4.00 (3.50–4.50)**	**3.25 (2.38–4.13)**	**0.013**	**0.345 (0.060 to 0.590)**	**Moderate**
SRS‐22 total score	3.82 (3.25–4.08)	3.50 (3.18–3.77)	0.158	0.197 (0.010 to 0.440)	Small
**BSSQ‐brace total score**	**9.00 (8.00–12.00)**	**7.50 (4.75–10.00)**	**0.007**	**0.376 (0.130 to 0.590)**	**Moderate**

*Note*: Data are presented as n (%) or median (IQR).

### QoL and Stress Levels in Men and Women

Between the male and female cohorts, no significant differences were noted in the measured variables, including function (*p* > 0.999), pain (*p* = 0.709), self‐image (*p* = 0.916), mental health (*p* = 0.658), treatment satisfaction (*p* = 0.628), SRS‐22 total score (*p* > 0.999), and BSSQ‐Brace total score (*p* = 0.657), with both the groups exhibiting comparable median values for each parameter. The effect sizes were small across all variables, with effects ranging from 0.002 to 0.069, indicating a minimal difference between male and female participants (Table 3).

**TABLE 3:** Comparison of QoL and stress levels between males and females.

Characteristic	Male N = 10	Female N = 42	*p*‐value^2^	Effect size (95%CI)	Magnitude
Function	3.90 (3.50–4.35)	4.00 (3.45–4.35)	>0.999	0.010 (0.006 to 0.330)	Small
Pain	3.90 (3.60–4.50)	3.90 (3.20–4.60)	0.709	0.053 (0.005 to 0.310)	Small
Self‐image	3.40 (2.65–3.75)	3.40 (2.85–3.80)	0.916	0.016 (0.003 to 0.300)	Small
Mental health	3.60 (2.70–3.95)	3.50 (2.85–4.20)	0.658	0.063 (0.004 to 0.330)	Small
Satisfaction	4.00 (3.13–4.00)	4.00 (3.13–4.50)	0.628	0.069 (0.005 to 0.300)	Small
SRS‐22 total score	3.64 (3.37–3.98)	3.64 (3.18–3.99)	>0.999	0.002 (0.004 to 0.290)	Small
BSSQ‐brace total score	9.00 (8.25–10.75)	9.00 (6.00–11.00)	0.657	0.063 (0.004 to 0.320)	Small

*Note*: Data are presented as n (%) or median (IQR).

### Subgroup Analysis of the Total SRS‐22 and BSSQ‐Brace Scores across Brace Wearing Times and Sex Categories

In the subgroup analysis of the total SRS‐22 and BSSQ‐brace scores, no significant differences were found in the SRS‐22 scores between the Boston and Chêneau braces in all duration categories (<6, <12, <24, and > 24 months, *P* = 0.540 within the Boston brace group and *p* = 0.334 within the Chêneau brace group). Similarly, no significant differences were observed for the BSSQ‐brace scores in the Boston brace group (*p* = 0.759) and the Chêneau brace group for all brace wearing time categories (*p* = 0.704).

For the Chêneau brace group, no significant differences were noted in SRS‐22 and BSSQ‐brace scores across the sex categories (*p* = 0.100 and *p* = 0.078, respectively). The same applies for differences between men and women in terms of the SRS‐22 score (*p* = 0.218) and the BSSQ‐brace score (*p* = 0.310) within the Boston brace group (Table 4).

**TABLE 4:** Subgroup analysis of the total SRS‐22 and BSSQ‐Brace scores across brace wearing times and sex categories

Parameter	Category	SRS‐22 score	*p*‐value	BSSQ‐brace score	*p*‐value
Brace	Boston				
	<6 m	3.32 (3.09–4.09)	0.540	9.00 (8.75–12.25)	0.759
	<12 m	4.02 (3.96–4.24)		8.50 (7.25–11.25)	
	<24 m	3.91 (3.61–4.05)		10.00 (8.75–11.25)	
	>24 m	3.63 (3.51–3.86)		11.00 (9.50–13.25)	
	Chêneau				
	<6 m	4.31 (3.81–4.54)	0.334	10.00 (8.00–10.00)	0.704
	<12 m	3.59 (3.50–3.64)		8.00 (6.00–8.00)	
	<24 m	3.04 (2.70–3.72)		7.00 (6.00–11.00)	
	>24 m	3.48 (3.21–3.62)		6.00 (3.75–8.50)	
Sex	Boston				
	Male	3.41 (3.09–3.84)	0.218	9.00 (8.00–10.00)	0.310
	Female	3.91 (3.27–4.18)		11.00 (8.00–12.00)	
	Chêneau				
	Male	3.90 (3.75–4.22)	0.100	10.00 (9.00–11.50)	0.078
	Female	3.36 (3.18–3.68)		6.00 (4.00–8.00)	

*Note*: Data are presented as median (IQR).

## Discussion

This study aimed to evaluate the differences in quality of life (QoL) and stress levels between patients with adolescent idiopathic scoliosis treated with Boston and Chêneau braces. The results indicate that while overall QoL as measured by the SRS‐22 Score did not differ significantly between the two brace groups, there were notable differences in specific subdomains.

### Satisfaction with Treatment

One of the most significant findings was that patients in the Boston brace group reported higher satisfaction compared to those in the Chêneau brace group. This could be linked to various factors including comfort, ease of use, or perceived effectiveness of the brace. The moderate effect size suggests that this is a meaningful difference likely to be of clinical importance. Previous literature on bracing for scoliosis has indicated that patient satisfaction can be influenced by brace design, with slimmer and less visible braces often preferred, which may partly explain our findings.^10^

### Stress Levels Associated with Brace Wear

Contrary to expectations, the Boston brace group reported higher scores on the BSSQ‐brace, indicating lower stress levels than those in the Chêneau brace group. This counterintuitive finding suggests that despite the potential for more conspicuous bracing with the Boston brace, patients may experience less stress, possibly due to factors such as better pain management, more effective communication regarding treatment, or other psychosocial factors not directly related to brace visibility.^22^, ^25^ The discrepancy between brace profile and stress levels warrants further investigation into how brace design, patient education, and psychosocial interventions contribute to the coping strategies employed by adolescents.^26^

### Gender Differences in QoL and Stress Levels

Our study did not find statistically significant differences between males and females in QoL or stress levels associated with brace wear. This suggests that gender may not play a major role in these psychosocial aspects of brace treatment for adolescent idiopathic scoliosis. These findings are consistent with some studies,^27^ but in contrast to others that have reported gender differences in the psychosocial impact of scoliosis treatment.^28^

### Impact of Brace Wearing Time

Subgroup analysis across different brace wearing times did not reveal statistically significant differences in QoL or stress levels for either brace type. This finding may indicate that the duration of brace wear does not exacerbate stress or diminish QoL over time, which is an encouraging sign for long‐term treatment adherence. However, it is important to consider that the lack of differences might also be due to the relatively small sample size, or the resilience developed by patients over time.^29^

### Limitations and Future Directions

This study has several limitations, including the cross‐sectional design, which allows for comparison at a single time point but not for tracking changes over the course of treatment. Additionally, the self‐reported nature of the SRS‐22 and BSSQ‐brace questionnaires, while valuable for capturing patient perspectives, may be subject to response biases. Also, the study did not utilize the Brace questionnaire (BRQ), which could provide further insights into the specific experiences and satisfaction of patients regarding brace treatment. Furthermore, the study did not consider the assessment of Risser sign, which is an important indicator of skeletal maturity in patients with scoliosis. Future research should aim to include larger, more diverse populations and consider longitudinal designs to assess changes in QoL and stress levels over time. It would also be beneficial to investigate the specific aspects of brace design and wear that most impact patient satisfaction and stress to guide improvements in brace construction and patient counseling.

## Conclusion

Overall, our study revealed that patients with AIS exhibited comparable QoL, regardless of the type of bracing used, based on the total scores from the SRS‐22r questionnaire. Nevertheless, individuals using the Chêneau brace reported higher stress levels and lower treatment satisfaction rates than those using the Boston brace. Furthermore, despite most AIS patients being women, sex did not appear to influence either the QoL or stress levels of patients. These results underscore the importance of further research on the use of AIS braces.

## Conflict of Interest Statement

The authors declare no competing interests.

## Ethics Statement

Informed consent was obtained from all participants and their legal guardian to participate in the study. Research has been performed in accordance with the Declaration of Helsinki and approved by institutional review board and ethical committee of College of Medicine, King Saud University, Riyadh, Saudi Arabia, under project number (IRB Project No. E‐23‐7548).

## Author Contributions

Abdulmonem Alsiddiky and Khalid AlHarbi conceived and designed the study. Khalid AlHarbi, Abdullah Alnuwaybit and Omar Ababtain collected the data and performed the analysis. Mazin Zamzami, Wesam Al‐Sabban and Ahmad Basalah assisted with the study. Khalid AlHarbi, Abdullah Alnuwaybit and Omar Ababtain wrote and revised the manuscript. All authors edited and approved the manuscript.
